# Nickel Hyperaccumulator Biochar as a Ni-Adsorbent and Enhanced Bio-ore

**DOI:** 10.1021/acsenvironau.1c00018

**Published:** 2021-10-07

**Authors:** Rachel A. Smoak, Jerald L. Schnoor

**Affiliations:** † Department of Civil and Environmental Engineering, University of Iowa, 4105 Seamans Center for the Engineering Arts and Sciences, Iowa City, Iowa 52242, United States; ‡ IIHR − Hydroscience and Engineering, University of Iowa, 100 C. Maxwell Stanley Hydraulics Laboratory, Iowa City, Iowa 52242, United States

**Keywords:** *Alyssum murale*, *Odontarrhena chalcidica*, hyperaccumulator, biochar, nickel, adsorption, bio-ore

## Abstract

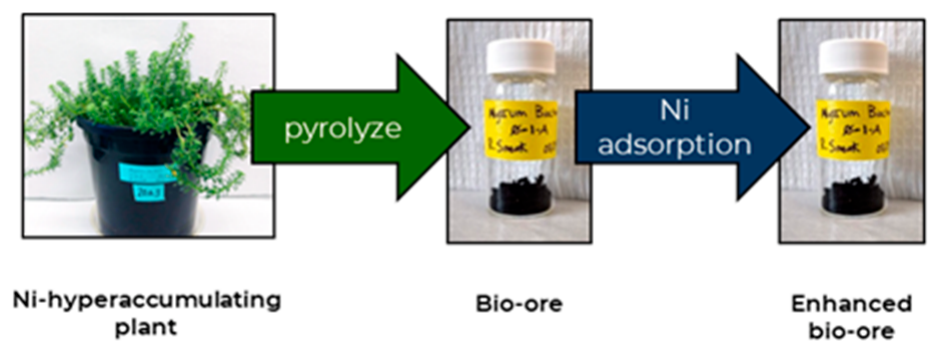

Increasing nickel (Ni) demand may spur the need for creative Ni production methods. Agromining (farming for metals) uses plants that can accumulate high concentrations of metal in their biomass, called bio-ore, as a metal extraction strategy. Furthermore, biochar, produced by biomass pyrolysis under low-oxygen conditions, can be used to remove Ni from contaminated wastewaters. In this work we investigate whether biochar synthesized from the Ni-hyperaccumulating plant *Odontarrhena chalcidica* (synonymous *Alyssum murale*) can be used as a Ni-adsorbing biochar. We grew *O. chalcidica* on soils with varying Ni concentration, characterized the plants and resultant biochars synthesized at different pyrolysis temperatures, and analyzed Ni batch adsorption results to determine the adsorption capacity of *O. chalcidica* biochar. We found that Ni concentration in *O. chalcidica* increases with increasing soil Ni but reaches an accumulation limit around 23 g Ni kg^–1^ dry weight in dried leaf samples. Pyrolysis concentrated Ni in the biochar; higher pyrolysis temperatures led to higher biochar Ni concentrations (max. 87 g Ni kg^–1^) and surface areas (max. 103 m^2^/g). Finally, the *O. chalcidica* biochar adsorption results were comparable to high-performing Ni adsorbents in the literature. The adsorption process greatly increased the Ni concentration in some biochars, indicating that synthesizing biochar from *O. chalcidica* biomass and using it as a Ni adsorbent can produce a Ni-enhanced bio-ore with nickel content higher than all nickel-rich veins currently mined.

## 1 Introduction

Steel is the most widely used metal in the world.^1^ Although steel is primarily composed of iron, nickel (Ni) is a primary component of making stainless steel. Ni is primarily extracted from magmatic sulfide or laterite mineral deposits.^2^ Most Ni production was historically from sulfide ores.^3^ Recently, an increasing proportion of Ni has been from laterite ores; Ni production, following rising Ni demand, has been consistently increasing since the 1930s.^2,4^ These trends are expected to continue, partially due to developing global infrastructure and Ni’s key role in electric vehicle battery production.^4,5^

Ideally, Ni recycling would balance Ni demand; however, projections indicate that higher primary Ni production will be required to meet demand.^4,5^ There is likely enough economically exploitable primary Ni to meet increased demand; however, mining, purifying, and refining Ni metal release greenhouse gases (7.64 kg CO_2_-eq/kg), degrade the environment (including soil contamination with heavy metals and acidification of local wetlands), and present human health concerns in nickel mining and refining workplaces.^2,3,6−11^ These negative effects of mining Ni (and all other mined resources) present a conflict of interest in societies increasingly devoted to combating climate change, environmental protection, sustainable development, and equitable global supply chains.^12^ The future of Ni production will be governed not only by resource availability and economic feasibility but also by environmental and social factors.^3,11^

An alternative method of primary Ni production which could complement mining lies in agromining, or “farming for metals”.^13^ Metal-hyperaccumulating plants uptake metal through low-selectivity cation transporters in the roots and, rather than pumping it out, store metals from the soil inside the plant biomass at concentrations higher than some commercially mined ores.^14^ The plants can then be sun-dried and incinerated to further concentrate the metal; the plant biomass also lacks high concentrations of major metal impurities found in conventional ore.^15−17^ Agromining cultivates these plants to recover metals from unmineable deposits in soils or mine tailings and then processes the metals into marketable products. Over 450 species of Ni-hyperaccumulating plants have been discovered. Agromining proponents have argued that Ni hyperaccumulators could be farmed, harvested, and introduced to the market as a new sustainable Ni source with fewer negative environmental impacts.^18,19^

New Ni hyperaccumulators, especially tropical species, are being consistently discovered, but the agronomy for some known temperate species is already well-established. One such species, *Odontarrhena chalcidica* (synonymous *Alyssum murale*), is a perennial originating in arid Mediterranean regions with serpentine soils including Turkey, Greece, and Albania, but it is able to grow and hyperaccumulate Ni outside its native environment.^20,21^*O. chalcidica* uptakes Ni primarily as Ni^2+^ in the roots, transports it to the leaves either in the hydrated ionic form or chelated with organic ligands, and stores Ni there.^22,23^ Adding fertilizer promotes biomass growth without lowering Ni concentration.^18,24^ Furthermore, adding organic soil amendments or cocropping legumes with *O. chalcidica* could eliminate the need for fertilizer in some circumstances, minimizing additional inputs to farm a metal crop.^25,26^ The product of a successful metal crop is known as a “bio-ore”. Ni recovery from a bio-ore typically involves ashing the plant material and subsequent hydrometallurgical and pyrometallurgical processes, although alternative processes can produce value-added Ni catalysts and chemical precursors.^19,27^

Biochar is a carbon-rich material produced by pyrolysis of a feedstock biomass under low-oxygen conditions. Several uses have been proposed for biochar, including as a soil amendment, a carbon sequestration method, and an adsorbent for environmental remediation.^28,29^ Biochars from many different feedstocks have demonstrated the ability to adsorb heavy metals, including Ni, from aqueous solutions.^30−33^ Ni pollution in water can originate from mining, metal refining, and industrial wastewater.^34,35^ Not only does Ni in wastewaters have negative environmental and human health impacts, but it also represents a lost commodity. After use, the adsorbent can contain high levels of heavy metal and must be disposed of or regenerated.

Although heavy-metal adsorbents have been derived from other hyperaccumulators, it appears that no Ni hyperaccumulator biochars have been tested as Ni adsorbents.^36^ We propose that biochar from *O. chalcidica* could be used to adsorb Ni from wastewater, becoming enhanced with higher nickel content, and then processed hydro- or pyro-metallurgically like a bio-ore. By increasing the Ni concentration in the biochar, one could increase its value as a metallurgical resource or even generate a valuable industrial material; various biochar-supported Ni structures are known to have favorable catalytic and electronic properties.^37−40^

To determine the viability of producing enhanced bio-ore, we grew *O. chalcidica* with varying soil Ni concentrations, synthesized and characterized biochar at varying pyrolysis temperatures, and measured the adsorption capacity of some resultant biochars. We found that the *O. chalcidica* biochar’s adsorption capacity is comparable to other adsorbents in the literature. Depending on the Ni concentration of the solution, the adsorption process can greatly increase the bio-ore’s Ni concentration, resulting in a Ni-enhanced bio-ore.

## 2 Experimental Section

### 2.1 Plant Growth and Characterization

**Figure 1:**
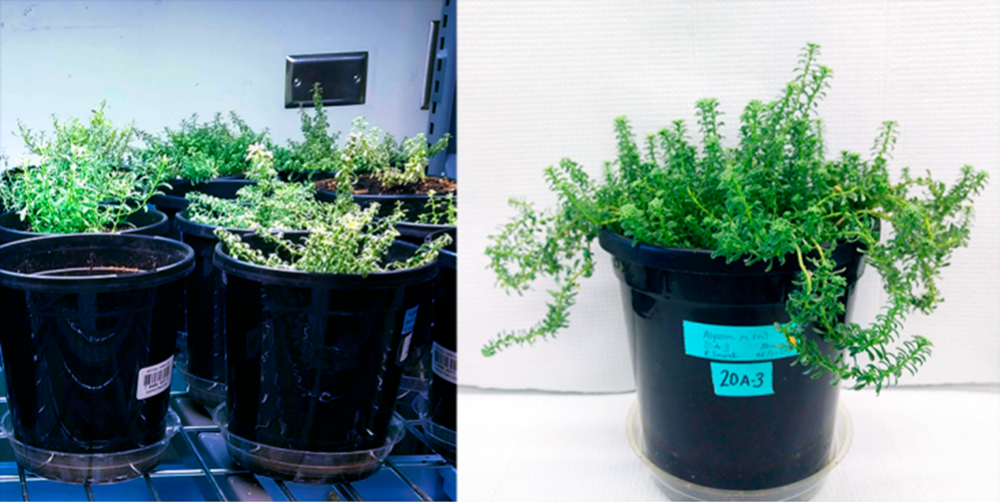
View of some *O. chalcidica* plants under the grow lights (left) and one of the whole shoot samples just prior to harvest (right).

*O. chalcidica* “Kotodesh” seeds (Albania, 1998) were obtained from the USDA in Beltsville, MD, and sprouted in nursery trays. Free-draining nursery tray cells were filled with ProMix potting soil, and seeds were pressed into the cells. Each cell was watered with 10 mL of RO water three times per week until plants sprouted in most cells. After reaching at least 2 cm in height, plants were transplanted into 1 gallon (3.8 L) freely drained plastic pots with saucers. The pots were each filled with 460 g of dry ProMix potting soil dosed with 0, 10, 20, 40, 60, 80, 100, 200, 300, 400, or 500 mmol Ni kg^–1^ (as NiSO_4_·6H_2_O, Fisher Scientific; approximately 0, 0.6, 1, 2, 4, 5, 6, 12, 18, 24, and 29 g Ni kg^–1^ dry potting mix, respectively) and mixed carbonates (half CaCO_3_ Fisher Scientific/half MgCO_3_ as C_4_H_2_Mg_5_O_14_·5H_2_O, Alfa Aesar) at concentrations equimolar with NiSO_4_.^41,42^ After dosing, soils in the pots were allowed to age for 1 month and rinsed with RO water to remove excess chemicals before *O. chalcidica* plants were introduced. The plants were watered twice per week with RO water to the point of soil saturation. Once every 2 weeks the plants were watered with a 1 tbsp of Miracle-Gro Water-Soluble All Purpose Plant Food:3.8 L RO water solution in place of simply RO water. The *O. chalcidica* pots were placed under grow lights (photosynthetically active radiation ∼280 μmol m^–1^s^–1^) with a 16/8 h on/off cycle and rotated weekly. They were allowed to grow for ∼6 months at room temperature, approximately 23 °C (Figure 1). After the plants were fully grown, the aerial portion of each plant was harvested and the leaves and stems were separated. The soil pH in each pot was also measured in triplicate according to U.S. EPA Method 9045D.^43^ The leaves and stems were dried in a drying oven at 105 °C for 1 week and weighed. The leaves were powdered with a mortar and pestle (referred to as leaf samples).

A second set of *O. chalcidica* plants were grown in the 0–80 mmol Ni kg^–1^ pots after the first plants were harvested for use as whole shoot plant samples. The growth procedure detailed above was used, the only exception being that the sprouts were transplanted into the previously used soils instead of newly aged soil. After ∼6 months the entire aerial portion of the plants was harvested and dried, and the soil pH was measured in triplicate. Stems and leaves were not separated, and the full aerial portion was powdered for further use (referred to as whole shoot samples).

Portable X-ray fluorescence spectroscopy (pXRF) was used to measure the metal, specifically Ni, content of the dried, powdered samples. An Olympus INNOV-X Delta Premium XRF analyzer in the soil analysis mode was used to measure 0.2 g of subsamples of plant material three times each for each plant with at least that much harvestable material. The subsamples were placed inside XRF sample cups (30.7 mm diameter, Chemplex Industries) and measured through transparent sample support windows (polypropylene, 0.24 mm thickness, Chemplex Industries). Because pXRF has only recently been applied to hyperaccumulators, a correlation for the pXRF measurements was built using the more common analytical method of inductively coupled plasma–optical emission spectrometry (ICP-OES).^44−47^ Specifically, representative leaf and whole shoot samples that covered the range of the pXRF-measured Ni content were ashed and acid digested according to WREP-125 Method B-4.10 and U.S. EPA Method 3050B.^48,49^ The digested samples were diluted and acidified in 4% HNO_3_ for ICP-OES analysis (Varian ICP-OES 720-ES). The ICP-OES measurements were assumed to be the “true” Ni concentrations, and all pXRF measurements were mathematically corrected using the correlation. Plant samples at the same Ni spike level with similar Ni concentrations were pooled, and the Ni contents of the pooled samples were measured. The bioaccumulation factor (BAF) is the prevalent metric used to determine how effectively a plant accumulates a species of interest from soil.^50^ We calculated the bioaccumulation factor for each of the pooled leaf samples by dividing the concentration of Ni in the leaves by the soil Ni spike (with the exception of the 0 mmol Ni kg^–1^ dry soil sample). After this, a one-way ANOVA with Tukey’s Pairwise Comparison test was performed on the pooled spike samples and the remaining unpooled samples. Samples that were not significantly different from each other were mixed into final leaf and whole shoot master mixes.

### 2.2 Biochar Pyrolysis and Characterization

Biochar was synthesized from the *O. chalcidica* samples in a Lindberg Blue M (Thermo Scientific) tube furnace. Three to five grams of plant sample were measured into each of two alumina crucibles, which were placed side-by-side in the tube furnace. The furnace was flushed with N_2_ (Praxair, 99.999% purity) for 1 h and 10 min at a flow rate of 20 sccm (N_2_ volume 1.5× tube furnace volume). The furnace was then heated to the desired pyrolysis temperature (400, 600, 750, or 900 °C) at 5 °C min^–1^ and held at that temperature for 1.5 h, after which it was allowed to cool to less than 50 °C naturally before the nitrogen flow was stopped. Given enough plant material, this procedure was repeated twice for each plant material/temperature combination, and the resultant biochar batches were mixed. Biochars were named for their parent plant material mix and pyrolysis temperature (plant mix-temperature).

Using the method described above, biochar samples were measured for the ICP-OES–pXRF correlation curve; however, no ashing was performed on the biochar. The Ni concentration of each biochar sample was analyzed with the described pXRF procedure. A pyrolysis concentration factor was calculated as the ratio of Ni in each biochar to its parent plant material. Performing a one-way ANOVA with pyrolysis temperature as a factor and a posthoc Tukey’s test resulted in an average concentration factor for each temperature and allows us to observe significant differences due to pyrolysis temperature.

The biochar surface area, structure, and surface elemental distribution were further characterized. Surface area was measured using a 6-point Brunauer–Emmett–Teller (BET) N_2_ adsorption method (Quantachrome Instruments 4200e) after vacuum drying each sample overnight at 105 °C. The surface structure and elemental distribution of selected biochars were characterized using scanning electron microscopy (SEM, Hitachi S-3400N) and energy dispersive X-ray spectroscopy (EDS, Bruker QUANTAX) after pressing a layer of biochar onto copper tape.

### 2.3 Biochar Ni^2+^ Adsorption

Batch adsorption experiments were performed to test the *O. chalcidica* biochar’s ability to adsorb Ni in an aqueous environment. Three biochars were selected for adsorption testing. Additionally, a granular activated carbon (GAC, Calgon Filtrasorb-200) was powdered and used as a reference material for the experiment. The inclusion of GAC in the experimental design allowed for a comparison against a well-characterized, commercially available biochar under controlled conditions. A 10 mM Ni stock solution was prepared from Ni(NO_3_)_2_·6H_2_O (Acros Organics). Appropriate amounts of the Ni stock were added to aliquots of a 0.01 M NaNO_3_ (Fisher Scientific) stock solution to obtain desired Ni^2+^ concentrations (0, 0.1, 0.2, 0.5, 1, 2, and 3 mM). The pH of each was adjusted to 5 using concentrated HNO_3_ and/or NaOH as needed. A pH of 5 was chosen to minimize nonsurface precipitation of Ni(OH)_2_ due to an increase of pH expected from the addition of an alkaline biochar and to allow pH-consistent comparison with literature adsorption values.^30,34^ A total of 0.05 g of adsorbent and 10 mL of diluted Ni solution were added to 20 mL glass scintillation vials so that each carbon–Ni solution combination was represented in duplicate. Negative controls of each Ni solution with no carbon were also made. The mixtures were shaken on a platform shaker at 200 rpm for 24 h and filtered with 0.45 μm Teflon filters; the pH of the filtrate was measured after ∼3 months of sealed, room temperature storage. The filtrates were diluted 1:1 in 4% HNO_3_ (VWR Chemicals) for ICP-OES analysis as above.^30^ The procedure was repeated for the biochar SHIGH-900 with a 6 mM Ni solution due to high Ni adsorption. The pH of each carbon material was also determined by placing 0.05 g into 10 mL of DI water, shaking on a platform shaker at 200 rpm for 24 h, allowing the carbon materials to settle, and measuring pH of the supernatant.

The amount of Ni adsorbed to each biochar sample was calculated using eq 1:
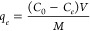
1where *q*_*e*_ (mmol/g) is the amount of Ni adsorbed on the biochar, *C*_0_ (mM) is the initial Ni concentration in the solution, *C*_*e*_ (mM) is the final Ni concentration in solution, *V* (L) is the solution volume, and *M* (g) is the mass of carbon in the vial. The percentage removal of Ni was calculated using eq 2:
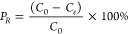
2where *P*_*R*_ is the percentage removal of Ni. Data were fitted using the Freundlich and Langmuir isotherm models, which are commonly used to describe adsorption isotherms.^51^ The empirically derived Freundlich isotherm can be written as
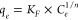
3where *K*_*F*_ (L^1/*n*^ mmol^(1–1/*n*)^/g) is a constant and 1/*n* is the Freundlich exponent. A linearized form is typically used to evaluate adsorption data and is written as
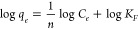
4The Langmuir isotherm assumes monolayer adsorption onto an adsorbent and can be written as
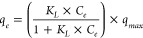
5where *K*_*L*_ (L/mmol) is a constant and *q*_*max*_ (mmol/g) relates to the theoretical maximum adsorption capacity of the adsorbent for the adsorbate. Calculations often use a linearized form:
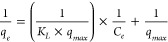
6

Negative control samples with no carbon were used as a proxy for initial Ni solution concentration. By doing so, we avoid attributing any systematic adsorption of Ni to the glass scintillation vial walls to the adsorbent.

Due to the toxicity of fumes containing Ni, the pyrolysis temperature should not be increased past the point where significant amounts of Ni are lost to the gas stream; appropriate safety precautions should be taken with the exit gas stream. In these experiments, exit gases were vented to outdoor air. Biochar should be treated as a toxic waste material, and care should be taken not to inhale powdered biochar samples since fine particulates and nickel are both carcinogenic.^9^

## 3 Results and Discussion

### 3.1 Plant Growth and Characterization

**Figure 2:**
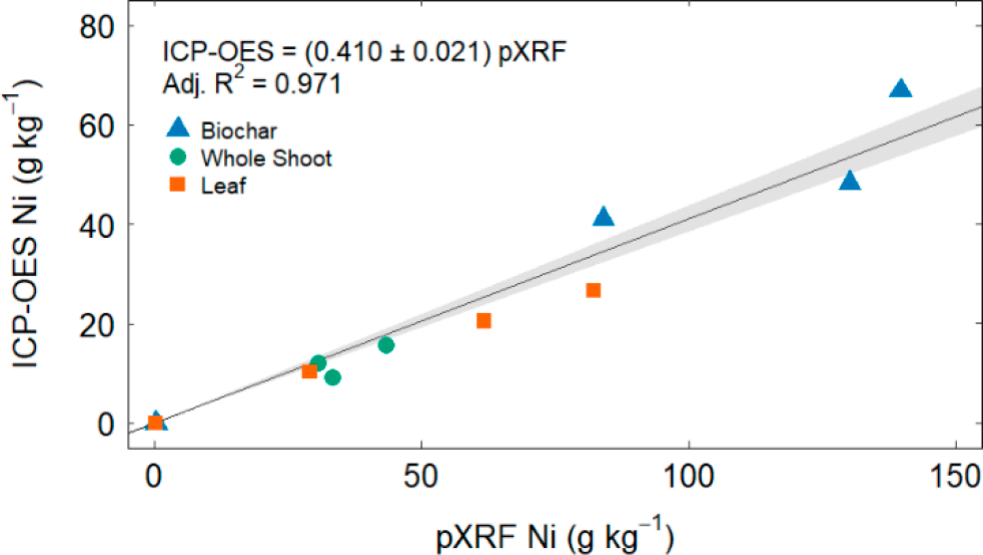
Curve correlating pXRF measurements of Ni in leaf, whole shoot, and biochar samples to ICP-OES measurements of the same samples. The measurements are pooled into one curve with a slope of 0.410. The shaded region is the 95% confidence interval.

The *O. chalcidica* plants showed no sign of phytotoxicity at any level of Ni dosing. Because both linear and power models have been used in the literature to fit pXRF–ICP-OES correlation curves, both models were applied to the data. The best-fitting model as determined by adjusted *R*^2^ and *p*-value was linear with a *y*-intercept of 0, although all models yield similar values over the range of interest (Figure 2). The pXRF and ICP-OES measurements were highly correlated, with a slope of 0.410 (adjusted *R*^2^ = 0.971, *p*-value = 2.95 × 10^–9^). The pXRF measurements tended to be approximately twice the true Ni content (as measured by ICP-OES) of the samples, perhaps because the low sample mass did not fully extinguish the X-rays. These results are consistent with results measuring other metals in plants, where pXRF and ICP-OES correlations can differ greatly by plant material and metal, demonstrating the necessity of measurement corrections.^52−54^ Other pXRF–ICP-OES curves for Ni in Ni hyperaccumulator plants provided extremely different numerical predictions from each other and from the correlation here given the same pXRF measurement, perhaps due to differences in pXRF instruments, measurement procedures, and species of plant measured.^45,47,55^ This demonstrates a continued need for instrument-, procedure-, and species-specific correlation curve development before uncorrected pXRF plant measurements should be used. pXRF measurements of metal content were much faster than ICP-OES measurements (∼10 min instead of 15 h per sample), and the high degree of correlation between the pXRF and ICP-OES measurements demonstrates that pXRF could be a very useful tool for hyperaccumulator metal analysis in the future.

**Figure 3:**
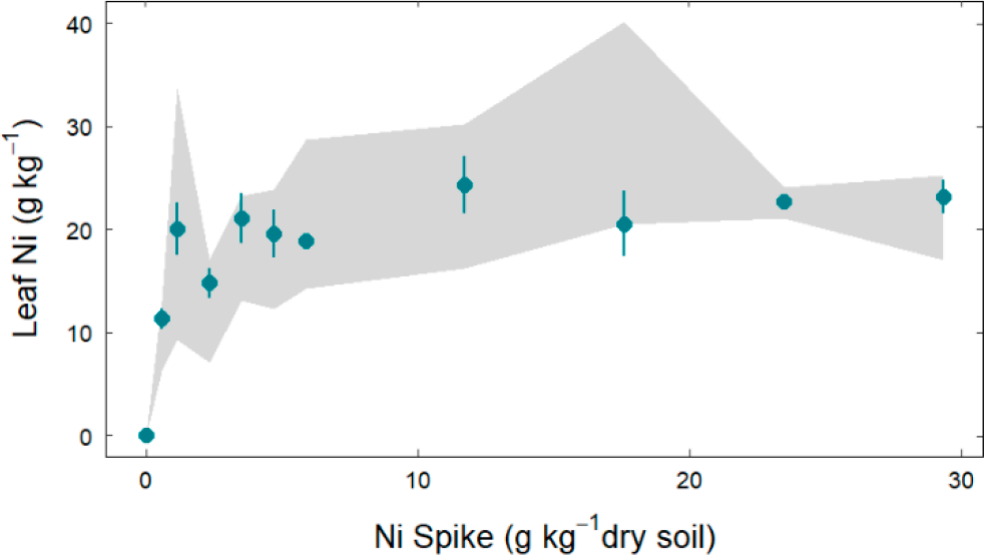
Dry weight leaf Ni measurements compared to the soil spike concentrations. The gray shaded region shows the range of measurements for individual plants at the given Ni spike concentration when there was sufficient material to measure; the points show the measurements after the leaves at each spike level that showed no significant difference in concentration were mixed. Error bars represent ± one standard deviation. (Error is within the marker where error bars are not visible.)

The Ni concentrations of each plant and soil pH after the experiment were measured individually and the Ni concentrations were mathematically corrected (Table S1 and Figure 3). We also measured the Ni concentrations of the pooled leaf samples (Figure 3 and Table S2) and combined them into the final master mixes (Table S3). The individual plant concentrations varied within the same dosing level, consistent with previous studies, indicating that the plant concentration of Ni could vary considerably even when grown in the same conditions.^41,42^ Dramatic concentration variations could prove problematic in commercializing this type of technology if Ni concentration needs to be tightly controlled; however, the statistically similar Ni concentrations of plants grown with different Ni spikes suggest that the average Ni concentration of a mixed plant batch may be maintained across different growth environments.

The threshold set to determine if a plant hyperaccumulates Ni is 1 g Ni kg^–1^ in the dried plant sample.^56^ All of the pooled leaf samples grown in Ni-spiked soil far surpassed this threshold; the minimum pooled leaf Ni concentration was 11.3 g Ni kg^–1^ at the soil spike of 10 mmol Ni kg^–1^ and the maximum pooled leaf Ni concentration was 24.3 g kg^–1^ at the soil spike of 200 mmol Ni kg^–1^ (see Table S2). The pooled leaf points in Figure 3 indicate that, generally, leaf Ni correlates positively with soil Ni until a saturation point is reached. The leaf Ni concentrations plateau at ∼23 g kg^–1^ (2.3 wt %). The data indicate that there is a limiting factor in the hyperaccumulation process and that Ni accumulation does not strictly increase with increasing soil Ni concentration; a saturation point exists.^41,42^ The mechanism underlying the Ni saturation has not been determined. Two hypotheses are that there could be a biological limit to Ni hyperaccumulation in *O. chalcidica* or that Ni hyperaccumulation was controlled by desorption of Ni from soil into pore water in our system.^57,58^ Modeling work on another Ni-hyperaccumulating plant suggests that in that case the Ni desorption rate from soil and the related plant transpiration rate were the controlling factors in plant Ni concentration, although in this case soluble Ni which one would expect to readily desorb was added to the potting mix.^59^ Regardless of the saturation mechanism, the concentration of Ni in the plant tissues rivals that of commercially mined Ni ores.^2^ Although estimating mined mineral resources can be challenging, one study reports that 90% of laterite ores and a similar percent of sulfide ores have a grade of <20 g kg^–1^ Ni; 23 g kg^–1^ certainly qualifies the *O. chalcidica* grown in this study as a rich bio-ore indeed.^3^

The pooled sample BAF values were calculated (Table S2). At the 10 mmol Ni kg^–1^ level, the BAF was 19.3, an extremely high BAF value. Any BAF > 1 demonstrates that the plant accumulates Ni from its surroundings. The pooled leaf samples with soil spikes 10–300 mmol Ni kg^–1^ all had BAF > 1. The BAF consistently decreased with an increase in the mass of Ni spiked, again demonstrating that leaf Ni concentration does not strictly increase with soil Ni concentration.

The Ni concentration results for the whole shoot samples show a similar trend to the results for the leaf samples (Table S2 and Figure S1). The pooled shoot Ni concentrations ranged between 12.6 and 17.8 g kg^–1^ at soil spikes of 10 and 80 mmol Ni kg^–1^, respectively; it plateaued at ∼16 g Ni kg^–1^ (1.6 wt %). Since a projected median concentration of Ni is 5 g kg^–1^ in mined sulfide ores and 11 g kg^–1^ in mined laterite ores, the whole shoot samples also qualify as a rich bio-ore.^3^ The BAF values for the pooled whole shoot samples ranged between 21.5 and 3.79, decreasing with increasing soil Ni spike. With the exception of the 60 mmol Ni kg^–1^ spike level, the whole shoot samples had comparable Ni concentrations to the leaf samples, even if the apparent concentration limit in the whole shoot trial was lower. The whole shoot trial only utilized the 0–80 mmol Ni pots, so an increase in whole shoot Ni concentration above that spike level is likely but was not tested experimentally.

### 3.2 Biochar Pyrolysis and Characterization

The Ni concentrations of the master mixes were measured (Table 1), and they were used to synthesize biochar under varying pyrolysis temperatures. The Ni concentrations of the resultant biochars were measured with pXRF and corrected using the ICP-OES correlation (Table 1). Overall, higher concentrations of Ni in the plant material led to higher concentrations of Ni in the biochar, as expected. A higher pyrolysis temperature also tended to lead to a higher biochar Ni concentration. This was not the case in all circumstances; SLOW-600 had an unexpectedly high Ni concentration, and there was no significant difference between SHIGH-400 and SHIGH-600. The overall effect of pyrolysis temperature, however, can be statistically examined. Table 2 shows that the pyrolysis concentration factor increased with increasing temperature. This is likely due to more complete decomposition of organics at higher temperatures without high Ni loss to the exiting gas stream.^60^ The mass of biochar and proportion of plant mass recovered as biochar decreased commensurately with the increase in pyrolysis temperature. The concentration factor at 400 °C is significantly different than that at 900 °C (*p* = 0.04); no other pair is significantly different. The increasing concentration factor with temperature indicates that we could potentially employ higher pyrolysis temperatures to further enhance biochar Ni concentration; however, we would expect a further reduction in biochar mass and more loss of Ni to the gas stream with increased pyrolysis temperature above 900 °C.^60^

**Table 1:** Plant Tissue Sample and Biochar Ni Concentrations

			biochar Ni^a^ (g kg^–1^)			
plant tissue	sample name	plant Ni (g kg^–1^)	400 °C	600 °C	750 °C	900 °C
leaf	L0	0.03 ± 0.01	-	0.16 ± 0.03	-	-
	LLOW	13.7 ± 1.5	-	38.9 ± 3.1	-	-
	LMED	21.5 ± 0.9	47.5 ± 3.1	57.7 ± 3.5	60.6 ± 2.5	-
	LHIGH	33.1 ± 2.0	75.6 ± 6.6	87.1 ± 11.9	-	-
whole shoot	S0	0.06 ± 0.01	0.16 ± 0.01	-	-	-
	SLOW	12.6 ± 2.1	27.4 ± 2.7	35.1 ± 1.8	33.0 ± 3.5	31.8 ± 4.5
	SHIGH	14.6 ± 1.0	36.2 ± 3.4	35.1 ± 3.4	41.6 ± 2.6	51.6 ± 3.7

a “-” indicates that there was no biochar for this plant material/temperature combination.

**Table 2:** Average Pyrolysis Temperature Concentration Factors (biochar Ni/plant Ni)

pyrolysis temperature (°C)	concentration factor
400	2.29 ± 0.15
600	2.67 ± 0.16
750	2.76 ± 0.20
900	3.03 ± 0.33

**Figure 4:**
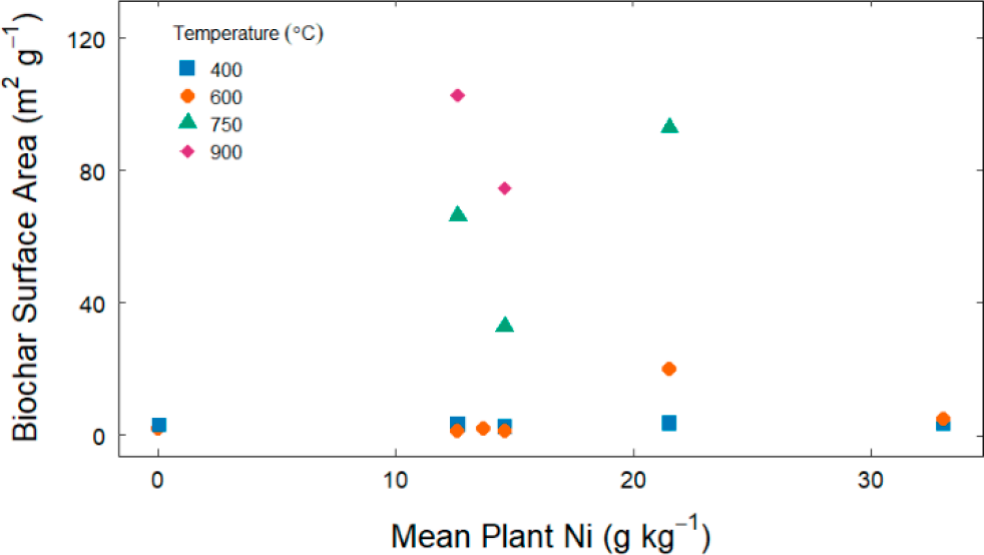
Biochar surface area as a function of mean plant Ni with marker shape/color indicating pyrolysis temperature. The biochar pyrolyzed at 400 or 600 °C has consistently and significantly lower surface area than that pyrolyzed at 750 or 900 °C.

The specific surface area of the biochars, as determined by BET, ranged between 1 and 103 m^2^/g (Figure 4 and Table S4). In general, biochar surface area increased with increasing pyrolysis temperature, as expected.^29^ An ANOVA analysis demonstrated that the effect of pyrolysis temperature on surface area was statistically significant (*p* < 0.01), although the effect of Ni concentration in the plant material was not. A Tukey’s test revealed that the biochars cluster by surface area into two significant groups: low temperature (400 and 600 °C) and high temperature (750 and 900 °C). The measured range of surface areas was within a typical range of surface area for unactivated biochar.^61^ Increased surface area is desirable for most biochar applications, so higher pyrolysis temperatures may be desirable. Other pyrolysis parameters such as heating rate, residence time, and carrier gas flow rate could be optimized to increase surface area, although only temperature was examined in this study. Surface area is also commonly increased through postpyrolysis chemical or physical activation. Taken together, concentration factor and surface area data both indicate that higher pyrolysis temperatures may be favorable.

The biochars chosen for SEM-EDS were LLOW-600, LHIGH-600, S0-400, SHIGH-400, SHIGH-600, SHIGH-750, and SHIGH-900. This allowed for at least one leaf-whole shoot, Ni concentration, and pyrolysis temperature comparison to be made. Overall, the biochar tended to look like a mixture of a small-grained powder and small monoliths up to 0.5 mm in size (Figures S2–S5). This size is similar to a tip-to-tip measurement of an intact *O. chalcidica* leaf trichome, although the observed monoliths seem more prismatic in shape.^41,62^ Initial Ni concentration seems to have no effect on structure (Figure S2). The biochars made from leaves only tend to have smaller and fewer monoliths than those made from the whole shoot (Figure S3). The fine structure of the leaf biochars is more plate-like while the whole shoot fine structure is more angular. This is likely the influence of stems in the whole shoot biochar, which do not powder as completely as the leaves. In general, gross structure of the biochar becomes more powder-like when the pyrolysis temperature exceeded 400 °C (Figure S4). The surface structure becomes more complex and pore structure becomes more open with increasing temperature; no evidence of thermal deactivation was observed.^61^ These phenomena could contribute to the higher surface area seen at increased pyrolysis temperature.

The EDS results showed that Ni was evenly dispersed across each sample and that potassium (K) and calcium (Ca) have strong signals in the EDS spectra of all samples (Figure S5). Although the EDS indicates that there may be significant amounts of K and Ca in the biochars, K and Ca are not measurable at the excitation wavelength used in the pXRF measurements and so were not quantified in this work. The presence of K and Ca is not unexpected; previous work has demonstrated that K and especially Ca are present in *O. chalcidica* leaves at elevated levels, with the Ca localized in the leaf trichomes in intact plants.^41,63^

### 3.3 Biochar Ni^2+^ Adsorption

**Figure 5:**
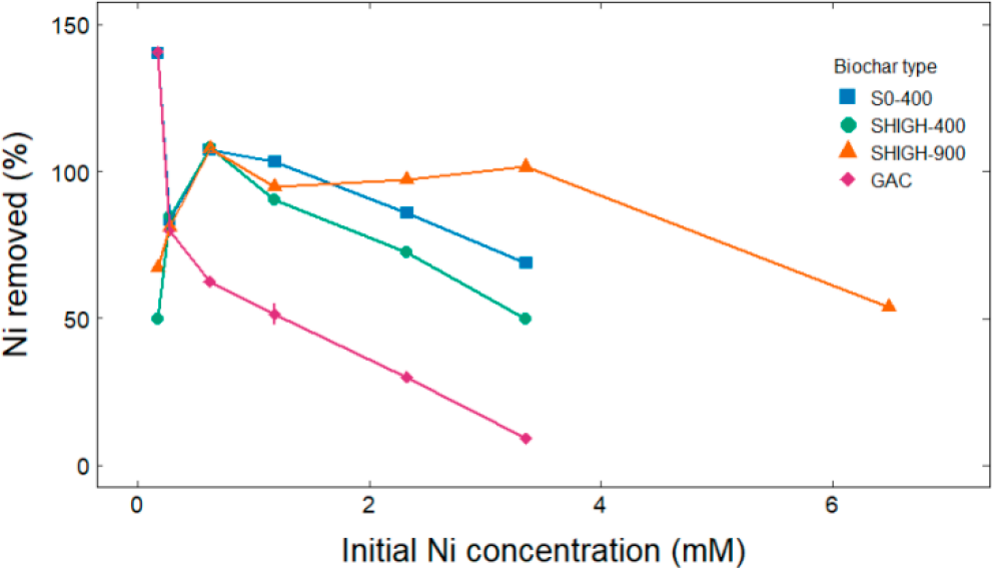
Percent of nickel removed from solution as a function of initial Ni concentration.

The Ni batch adsorption experiments were carried out using biochars S0-400, SHIGH-400, SHIGH-900, and the reference material GAC. Carbon pH is reported in Table 3. In addition to the adsorption experiment biochars, LHIGH-600, the highest Ni biochar, was subjected to the 0 mM Ni treatment under the same conditions (Table S5). No significant amount of Ni was detected in any 0 mM Ni solution after the experiment, likely due to the alkalinity of both the biochars and resultant equilibrium solutions; leaching is conducted under acidic conditions.^27^ Filtrate pH ranged between 7.1 and 11.8 with lower pH at higher Ni concentrations, as expected due to release of more H^+^ with higher metal ion uptake.^34^ The amount of Ni adsorbed to each adsorbent was calculated using eq 1, and the percent of Ni removed was calculated using eq 2 (Figure 5). Removal of over 100% of the Ni is an artifact of the measurement; low concentrations of Ni have higher relative measurement error, and this is propagated through the calculations. Any removal value of over 100% should be understood as ∼100% removal.

**Table 3:** Maximum Observed Specific Adsorption of Ni^2+^

adsorbent	pH	observed *q*_*max*_ (mg Ni/g adsorbent)
S0-400	10.5	27.2 ± 0.5
SHIGH-400	10.4	19.9 ± 0.1
SHIGH-900	10.4	40.8 ± 1.3
GAC	7.48	8.16 ± 0.54

The GAC showed a decreasing Ni removal efficiency typical of Langmuir or Freundlich adsorption patterns.^64^ Removal efficiency consistently decreased with increasing Ni concentration, and above 0.2 mM Ni GAC had the lowest Ni removal efficiency of any adsorbent. S0-400 and SHIGH-400 showed similar Ni removal patterns. Both showed peak removal in the 0.5 mM Ni solution, where they removed ∼100% of the Ni. Above 0.5 mM Ni, Ni removal consistently decreased with increasing initial concentration, although S0-400 outperformed SHIGH-400 at all points. S0-400’s slightly higher surface area could have contributed to its better performance. Above the 0.5 mM Ni concentration, SHIGH-900 consistently removed ∼100% of Ni until the 6 mM solution point, where it reached its adsorption maximum. The biochars all outperformed the commercially available GAC for solutions with Ni concentrations ≥ 0.5 mM. The strong alkalinity of the biochars compared to the GAC could aid in Ni adsorption due to surface precipitation of salts.^30^ The biochars all had similar pH values; their differences in adsorption capacity trended with and could be attributable to higher surface area, which could provide more adsorption sites.^32^

Freundlich and Langmuir isotherms were fitted to the GAC, S0-400, and SHIGH-400 data (Table S6). Solution concentrations of less than 0 were ignored in the calculations since they are not physically feasible. SHIGH-900 was not fitted to an isotherm model because the data only contain Ni in solution at equilibrium in the 6 mM starting solution, making the calculations inappropriate. The adsorption points and better-fitting isotherms as determined by *R*^2^ values are shown in Figure S6. Although adsorption capacities are specific to experimental conditions such as metal mix in solution, temperature, and pH, these results suggest that the maximum observed adsorptions (Table 3) are comparable to other promising adsorbents in the literature, which range between 1 and 90 mg Ni g^–1^.^34,65,66^ Of the 45 Ni adsorbents represented in these studies, only 11 have a higher maximum adsorption capacity than the observed adsorption of the *O. chalcidica* biochar. This is a promising finding, especially since the adsorption capacity of all *O. chalcidica* biochars may further improve with activation of the biochar.

One problem with metal adsorbents is disposal after use. The *O. chalcidica* biochars already contained significant amounts of Ni, which was enhanced through the adsorption of more Ni from solution. An estimate of the final amount of Ni in the biochars after adsorption can be obtained by adding the initial concentration to the adsorbed concentration (Table S7). The adsorbed Ni can be greater than or equal to the initial Ni in the biochar, significantly increasing Ni in the final biochar. For example, after adsorption from the 3 mM Ni solution S0-400 and SHIGH-900 went from 0.16 and 52 g Ni kg^–1^ to 27 and 92 g Ni kg^–1^, respectively. Most Ni hyperaccumulator bio-ores contain 10–60 g Ni kg^–1^ before Ni extraction processing, depending on their species and growing conditions.^24^ Adsorption increased the biochar Ni concentration by up to 41 g kg^–1^, which is more Ni than many bio-ores natively contain. Postadsorption biochar could then be used as an enhanced bio-ore in a typical Ni hyperaccumulator Ni extraction process, circumventing the need to develop an additional regeneration or disposal process.

## 4 Conclusion

This study demonstrates the potential use of biochar from the Ni hyperaccumulator plant *O. chalcidica* as a Ni adsorbent to synthesize an enhanced bio-ore. To the best of our knowledge, it is the first time that Ni hyperaccumulator plant biomass has been used to prepare a unique biochar and examined for Ni adsorptive properties. We have shown that correlated pXRF measurements can be used as a quick and easy Ni measurement in plant material and biochar. The Ni concentration of *O. chalcidica* biomass varies according to the Ni concentration in soil but has an upper limit likely depending on either a hyperaccumulation limit in the plant or a limiting Ni desorption rate from the soil into pore water. The upper limit of 16 g kg^–1^ in whole shoot samples exceeded the median concentration of mined Ni ore deposits; the upper limit of 23 g Ni kg^–1^ in leaf samples is comparable to the 90th percentile concentration of ore deposits worldwide.

The Ni concentration in *O. chalcidica* biochar increased with both initial plant material Ni concentration and pyrolysis temperature; increased pyrolysis temperature also increased the BET surface area of the biochar. Ni adsorption experiments demonstrated that *O. chalcidica* biochar outperformed commercially available GAC, potentially due to the biochar’s strong alkalinity. The observed Ni adsorption was comparable to high-performing, activated biochars in the literature, indicating an opportunity to use *O. chalcidica* biochar as an adsorbent material in high-Ni wastewaters.

The main proposed use of Ni-hyperaccumulating plants is as a bio-ore for Ni production. High Ni content is very important in bio-ores. The postadsorption *O. chalcidica* biochar significantly increased in Ni concentration and could potentially be used as an enhanced bio-ore in the fledgling Ni hyperaccumulator Ni production industry. Future work should optimize the biochar for Ni adsorption, determine metal adsorption in systems that mimic wastewaters, determine the geographic and economic feasibility of using the biochar as an adsorbent, test processing methods for the enhanced bio-ore, and investigate other catalytic and energy storage applications of enhanced bio-ore. Using both *O. chalcidica*’s metal and biomass as a resource could be a green engineering solution to address sustainability and ethical considerations of Ni production and removal of Ni from high-Ni wastewater while simultaneously providing a high-Ni enriched bio-ore.

## Supporting Information Available

Additional numeric results, master mix information, photographs of experimental setup, whole shoot Ni results, adsorption isotherms, and SEM-EDS results (PDF)

## Notes

The underlying data for this work has been deposited in the Iowa Research Online (IRO) institutional data repository for future reuse under an Open Data Commons Attribution License (ODC-By).^67^ There are no registration or fee requirements to download the underlying data set for this work.

## Abbreviations

BAF: bioaccumulation factor
BET: Brunauer–Emmett–Teller
C: carbon
Ca: calcium
GAC: granular activated carbon
ICP-OES: inductively coupled plasma–optical emission spectrometry
K: potassium
Ni: nickel
pXRF: portable X-ray fluorescence spectroscopy
SEM-EDS: scanning electron microscopy–energy dispersive X-ray spectroscopy
