# Supplementary material for: Nickel Hyperaccumulator Biochar as a Ni-Adsorbent and Enhanced Bio-ore
Source: ACS Environ Au. 2021 Oct 7;2(1):65–73. doi: 10.1021/acsenvironau.1c00018 (PMC8778606; doi:10.1021/acsenvironau.1c00018)
Supplement: Supplementary file 1 — vg1c00018_si_001.pdf [file vg1c00018_si_001.pdf]

Supporting Information

Nickel hyperaccumulator biochar as a Ni-adsorbent and enhanced bio-ore

Rachel A. Smoak*†‡, Jerald L. Schnoor†‡

†Department of Civil and Environmental Engineering, University of Iowa, 4105 Seamans Center for the Engineering Arts and Sciences, Iowa City, Iowa 52242, USA

‡IIHR – Hydroscience and Engineering, University of Iowa, 100 C. Maxwell Stanley Hydraulics Laboratory, Iowa City, Iowa, 52242, USA

Table S1. Individual plant Ni concentrations expressed per kilogram of dry weight

	Initial Soil Spike (mmol/kg)	Sample Name	Plant Ni (g kg^{-1} dry weight)	Soil pH
**Leaf**	0	0-A	0.03 ± 0.00	6.25 ± 0.10
		0-B	-	6.34 ± 0.05
		0-C	0.01 ± 0.00	6.16 ± 0.04
		0-D	0.03 ± 0.00	6.17 ± 0.04
		0-E	0.08 ± 0.01	5.65 ± 0.07
		0-F	0.05 ± 0.02	5.41 ± 0.13
		0-G	0.05 ± 0.01	5.52 ± 0.04
		0-H	-	5.75 ± 0.03
	10	10-A	6.33 ± 0.02	6.32 ± 0.06
		10-B	8.98 ± 0.04	6.33 ± 0.08
		10-C	12.4 ± 0.1	6.52 ± 0.01
		10-D	13.0 ± 0.1	6.64 ± 0.10
	20	20-A	12.7 ± 0.1	6.54 ± 0.06
		20-B	33.7 ± 0.3	6.70 ± 0.12
		20-C	15.2 ± 0.3	6.59 ± 0.12
		20-D	13.5 ± 0.1	6.66 ± 0.15
		20-E	9.24 ± 0.10	7.00 ± 0.05
	40	40-A	17.1 ± 0.1	6.51 ± 0.09
		40-B	13.5 ± 0.1	6.56 ± 0.11
		40-C	14.7 ± 0.1	6.93 ± 0.08
		40-D	7.07 ± 0.09	6.62 ± 0.11
	60	60-A	22.2 ± 0.1	6.74 ± 0.07
		60-B	23.3 ± 0.1	6.91 ± 0.03
		60-C	13.3 ± 0.0	6.98 ± 0.06
		60-D	13.1 ± 0.1	6.85 ± 0.09
	80	80-A	16.2 ± 0.1	7.24 ± 0.08
		80-B	-	7.26 ± 0.07
		80-C	23.8 ± 0.1	7.29 ± 0.07
		80-D	12.3 ± 0.1	7.05 ± 0.06
	100	100-A	15.6 ± 0.8	6.65 ± 0.03
		100-B	14.3 ± 0.5	6.31 ± 0.16
		100-C	19.6 ± 1.2	6.48 ± 0.09
		100-D*	28.7 ± 2.6	6.76 ± 0.06
	200	200-A*	30.2 ± 3.6	7.73 ± 0.11
		200-B	22.3 ± 2.2	7.54 ± 0.22
		200-C	16.2 ± 1.3	7.88 ± 0.07
		200-D*	29.3 ± 3.6	7.85 ± 0.06
	300	300-A*	40.1 ± 2.9	8.26 ± 0.10
		300-B	-	8.46 ± 0.10

	Initial Soil Spike (mmol/kg)	Sample Name	Plant Ni (g kg^{-1} dry weight)	Soil pH
		300-C	-	8.44 ± 0.11
		300-D	23.7 ± 0.8	8.37 ± 0.04
	400	400-A	21.1 ± 2.4	8.70 ± 0.05
		400-B	-	8.73 ± 0.04
		400-C	24.1 ± 1.6	8.68 ± 0.05
		400-D	-	8.47 ± 0.02
	500	500-A	19.6 ± 1.1	8.54 ± 0.04
		500-B	25.3 ± 1.2	8.56 ± 0.10
		500-C	17.1 ± 1.2	8.37 ± 0.02
		500-D	-	8.33 ± 0.06
**Whole Shoot**	0	0-A	0.05 ± 0.01	6.25 ± 0.10
		0-B	0.05 ± 0.00	6.34 ± 0.05
		0-C	0.08 ± 0.01	6.16 ± 0.04
		0-D	0.09 ± 0.01	6.17 ± 0.04
	10	10-A	11.8 ± 0.7	6.32 ± 0.06
		10-B	11.1 ± 1.1	6.33 ± 0.08
		10-C	14.5 ± 1.1	6.52 ± 0.01
		10-D	16.8 ± 2.1	6.64 ± 0.10
	20	20-A	15.7 ± 1.3	6.54 ± 0.06
		20-B	16.4 ± 1.8	6.70 ± 0.12
		20-C	12.4 ± 1.8	6.59 ± 0.12
		20-D	14.4 ± 1.0	6.66 ± 0.15
		20-E	22.0 ± 1.9	7.00 ± 0.05
	40	40-A	11.9 ± 0.5	6.51 ± 0.09
		40-B	10.3 ± 1.0	6.56 ± 0.11
		40-C	23.9 ± 1.6	6.93 ± 0.08
		40-D	11.7 ± 1.6	6.62 ± 0.11
	60	60-A	12.3 ± 0.4	6.74 ± 0.07
		60-B	11.1 ± 1.0	6.91 ± 0.03
		60-C	10.4 ± 0.5	6.98 ± 0.06
		60-D	16.5 ± 1.6	6.85 ± 0.09
	80	80-A	19.3 ± 1.4	7.24 ± 0.08
		80-B	19.9 ± 2.0	7.26 ± 0.07
		80-C	19.0 ± 1.6	7.29 ± 0.07
		80-D	16.9 ± 0.8	7.05 ± 0.06

- indicates that there was not enough harvestable plant material to measure; * indicates that the sample was withheld from the pooled spike level mix

Table S2. Pooled leaf and whole shoot Ni concentrations and biological accumulation factors (BAF)

	Initial Soil Spike (mmol Ni kg^{-1} dry soil)	Initial Soil Spike (g Ni kg^{-1} dry soil)	Plant Ni (g Ni kg^{-1} dry weight)	BAF
**Leaf**	10	0.59	11.3 ± 1.0	19.3 ± 1.7
	20	1.17	20.1 ± 2.5	17.1 ± 2.2
	40	2.35	14.8 ± 1.4	6.31 ± 0.61
	60	3.52	21.1 ± 2.4	6.00 ± 0.69
	80	4.70	19.6 ± 2.3	4.18 ± 0.49
	100	5.87	18.9 ± 0.6	3.21 ± 0.11
	200	11.7	24.3 ± 2.8	2.07 ± 0.24
	300	17.6	20.6 ± 3.2	1.17 ± 0.18
	400	23.5	22.7 ± 0.5	0.97 ± 0.02
	500	29.3	23.1 ± 1.6	0.79 ± 0.05
**Whole Shoot**	10	0.59	12.6 ± 2.1	21.5 ± 3.6
	20	1.17	16.4 ± 0.9	14.0 ± 0.8
	40	2.35	15.2 ± 2.4	6.48 ± 1.03
	60	3.52	13.7 ± 1.9	3.88 ± 0.54
	80	4.70	17.8 ± 2.5	3.79 ± 0.53

Table S3. Samples mixed to make master mixes

	Mix Name	Samples Mixed
**Leaf**	L0	0-A, 0-C, 0-C, 0-E, 0-F, 0-G
	LLOW	10-A, 10-B, 10-C, 10-D, 40-A, 40-B, 40-C, 40-D
	LMED	20-A, 20-B, 20-C, 20-D, 20-E, 60-A, 60-B, 60-C, 60-D, 80-A, 80-C, 80-D, 100-A, 100-B, 100-C, 200-B, 200-C, 300-D, 400-A, 400-C, 500-A, 500-B, 500-C
	LHIGH	100-D, 200-A, 200-D, 300-A
**Whole Shoot**	S0	0-A, 0-B, 0-C, 0-D
	SLOW	10-A, 10-B, 10-C, 10-D, 60-A, 60-B, 60-C, 60-D
	SHIGH	20-A, 20-B, 20-C, 20-D, 20-E, 40-A, 40-B, 40-C, 40-D, 80-A, 80-B, 80-C, 80-D

Table S4. BET surface area measurements for all biochars

	Sample Name	BET Surface Area (m^2/g)			
		**400°C**	**600°C**	**750°C**	**900°C**
**Leaf**	L0	-	2.11	-	-
	LLOW	-	2.07	-	-
	LMED	3.49	19.9	92.9	-
	LHIGH	3.31	4.66	-	-
**Whole Shoot**	S0	2.93	-	-	-
	SLOW	3.16	1.32	66.2	103
	SHIGH	2.48	1.09	32.8	74.7

- indicates that there was no biochar synthesized at the indicated sample/temperature combination

Table S5. Adsorption measurements for biochars

Biochar	Biochar mass (mg)	Ni solution (nominal mM)	Final Ni solution concentration (mM)	Final filtered Ni solution pH*
S0-400	50.0 ± 0.5	0	-0.0716 ± 0.0003	8.80 ± 0.37
	50.1 ± 0.3	0.1	-0.0714 ± 0.0004	8.57 ± 0.04
	50.1 ± 0.1	0.2	0.0464 ± 0.0005	8.54 ± 0.08
	50.0 ± 0.2	0.5	-0.0460 ± 0.0006	8.68 ± 0.19
	49.8 ± 0.0	1	-0.0403 ± 0.0006	8.29 ± 0.11
	49.9 ± 0.2	2	0.331 ± 0.008	8.31 ± 0.10
	50.0 ± 0.1	3	1.04 ± 0.02	8.09 ± 0.01
SHIGH-400	50.1 ± 0.1	0	-0.0732 ± 0.0003	8.36 ± 0.03
	50.0 ± 0.1	0.1	0.0889 ± 0.0006	8.34 ± 0.24
	50.0 ± 0.3	0.2	0.0432 ± 0.0003	8.41 ± 0.01
	49.9 ± 0.3	0.5	-0.0518 ± 0.0005	8.27 ± 0.12
	49.8 ± 0.1	1	0.117 ± 0.001	8.26 ± 0.12
	49.7 ± 0.1	2	0.643 ± 0.006	7.96 ± 0.18
	50.1 ± 0.6	3	1.68 ± 0.01	7.64 ± 0.23
SHIGH-900	50.2 ± 0.0	0	-0.0610 ± 0.0008	11.6 ± 0.3
	50.1 ± 0.1	0.1	0.0580 ± 0.0008	11.5 ± 0.4
	50.2 ± 0.5	0.2	0.0532 ± 0.0004	11.8 ± 0.1
	50.2 ± 0.3	0.5	-0.0480 ± 0.0005	11.5 ± 0.5
	50.0 ± 0.3	1	0.0598 ± 0.0003	11.8 ± 0.0
	50.2 ± 0.2	2	0.0636 ± 0.0005	9.98 ± 2.38
	49.8 ± 0.1	3	-0.0508 ± 0.0002	9.50 ± 2.21
	50.0 ± 0.2	6	3.00 ± 0.10	7.84 ± 0.03
GAC	50.7 ± 0.1	0	-0.0421 ± 0.0003	7.48 ± 0.45
	50.3 ± 0.6	0.1	-0.0724 ± 0.0003	7.51 ± 0.28
	50.2 ± 0.4	0.2	0.0566 ± 0.0004	7.40 ± 0.06
	48.4 ± 2.2	0.5	0.234 ± 0.004	7.14 ± 0.05
	49.8 ± 0.2	1	0.576 ± 0.045	7.26 ± 0.07
	50.0 ± 0.1	2	1.63 ± 0.05	7.10 ± 0.06
	50.0 ± 0.2	3	3.05 ± 0.04	6.92 ± 0.30
BLANK		0	-0.0588 ± 0.0005	6.94 ± 0.01
		0.1	0.177 ± 0.001	6.93 ± 0.56
		0.2	0.279 ± 0.004	6.84 ± 0.53
		0.5	0.624 ± 0.002	6.57 ± 0.40
		1	1.19 ± 0.01	6.96 ± 0.35
		2	2.33 ± 0.01	6.45 ± 0.62
		3	3.35 ± 0.06	5.48 ± 1.40
		6	6.48 ± 0.04	6.55 ± 0.04
LHIGH-600	50.1 ± 0.3	0	-0.0580 ± 0.0006	8.62 ± 0.26

BLANK samples had no biochar added; they were used as a proxy of initial concentration minus any Ni sorbed to tube walls (which seemed minimal), * pH measurements were taken after ~3 months sealed, room-temperature storage

Table S6. Freundlich and Langmuir sorption isotherm fitted parameters

Isotherm model	Parameter (dimensions)	S0-400	SHIGH-400	GAC
Freundlich	K_F ($L^{1/n}mmol^{(1-1/n)}/g$)<br>$1/n$ (-)	0.598<br>0.777<br>(r^2 = 0.902)	0.317<br>0.664<br>(r^2 = 0.577)	0.0905<br>0.149<br>(r^2 = 0.246)
Langmuir	K_L (L/mmol)<br>q_{max} (mmol/g)	0.443<br>2.31<br>(r^2 = 0.994)	6.57<br>0.134<br>(r^2 = 0.200)	14.4<br>0.100<br>(r^2 = 0.634)

Table S7. Biochar adsorbed Ni and estimated final Ni concentrations

Biochar	Biochar initial Ni ($g\ kg^{-1}$)	Ni solution (nominal mM)	Biochar sorbed Ni ($g\ kg^{-1}$)	Estimated biochar final Ni ($g\ kg^{-1}$)
S0-400	0.16 ± 0.01	0	0.150 ± 0.005	0.31 ± 0.01
		0.1	2.91 ± 0.01	3.08 ± 0.01
		0.2	2.73 ± 0.03	2.89 ± 0.03
		0.5	7.87 ± 0.02	8.03 ± 0.02
		1	14.5 ± 0.1	14.6 ± 0.1
		2	23.5 ± 0.1	23.7 ± 0.1
		3	27.2 ± 0.5	27.3 ± 0.5
SHIGH-400	36.2 ± 3.4	0	0.169 ± 0.005	36.3 ± 3.4
		0.1	1.04 ± 0.01	37.2 ± 3.4
		0.2	2.77 ± 0.03	38.9 ± 3.4
		0.5	7.95 ± 0.02	44.1 ± 3.4
		1	12.6 ± 0.1	48.8 ± 3.4
		2	19.9 ± 0.1	56.1 ± 3.4
		3	19.6 ± 0.5	55.8 ± 3.4
SHIGH-900	51.6 ± 3.7	0	0.025 ± 0.010	51.6 ± 3.7
		0.1	1.40 ± 0.01	53.0 ± 3.7
		0.2	2.65 ± 0.03	54.2 ± 3.7
		0.5	7.86 ± 0.02	59.5 ± 3.7
		1	13.2 ± 0.1	64.8 ± 3.7
		2	26.5 ± 0.1	78.1 ± 3.7
		3	40.1 ± 0.5	91.7 ± 3.7
		6	40.8 ± 1.3	92.4 ± 3.9

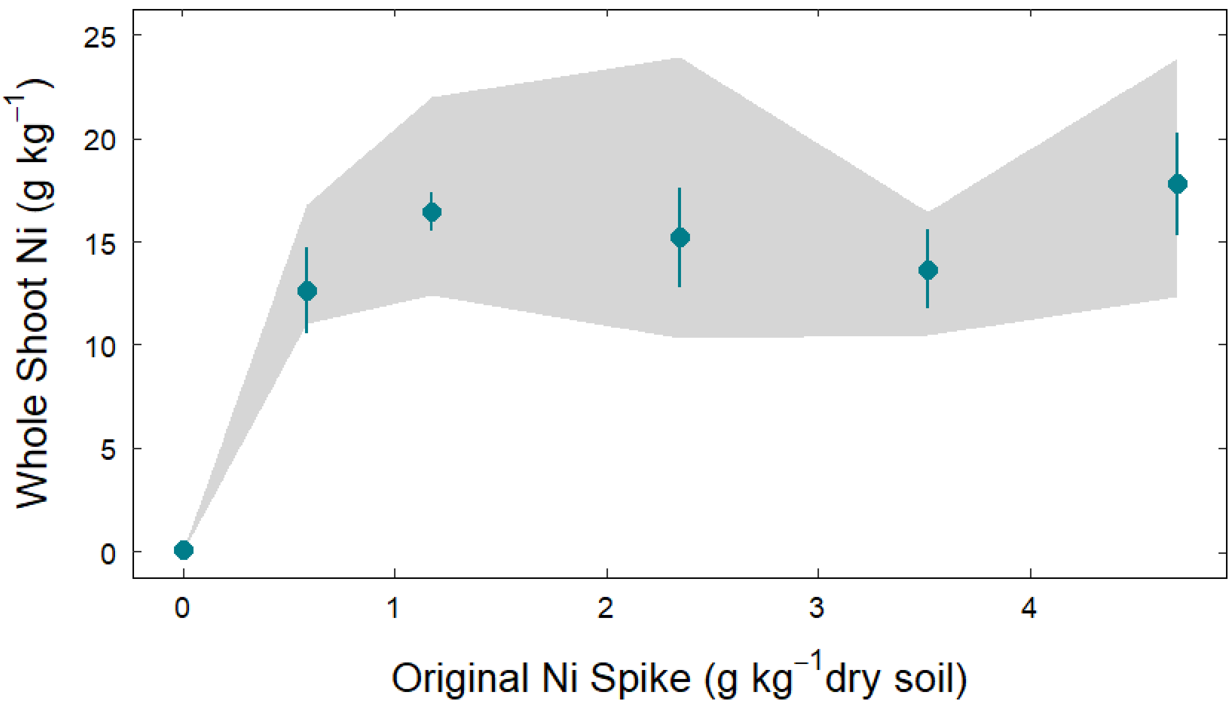

Figure S1. The whole shoot Ni measurements compared to the original soil spike concentrations. The gray shaded region shows the range of measurements for individual plants at the given Ni concentration when there was sufficient material to measure; the points show the measurements after the whole shoot samples at each spike level were mixed. Error bars represent ± one standard deviation. Error is within the marker where error bars are not visible. Ni concentration increased between 0 and 1 g kg^{-1} dry soil but was relatively consistent above that.

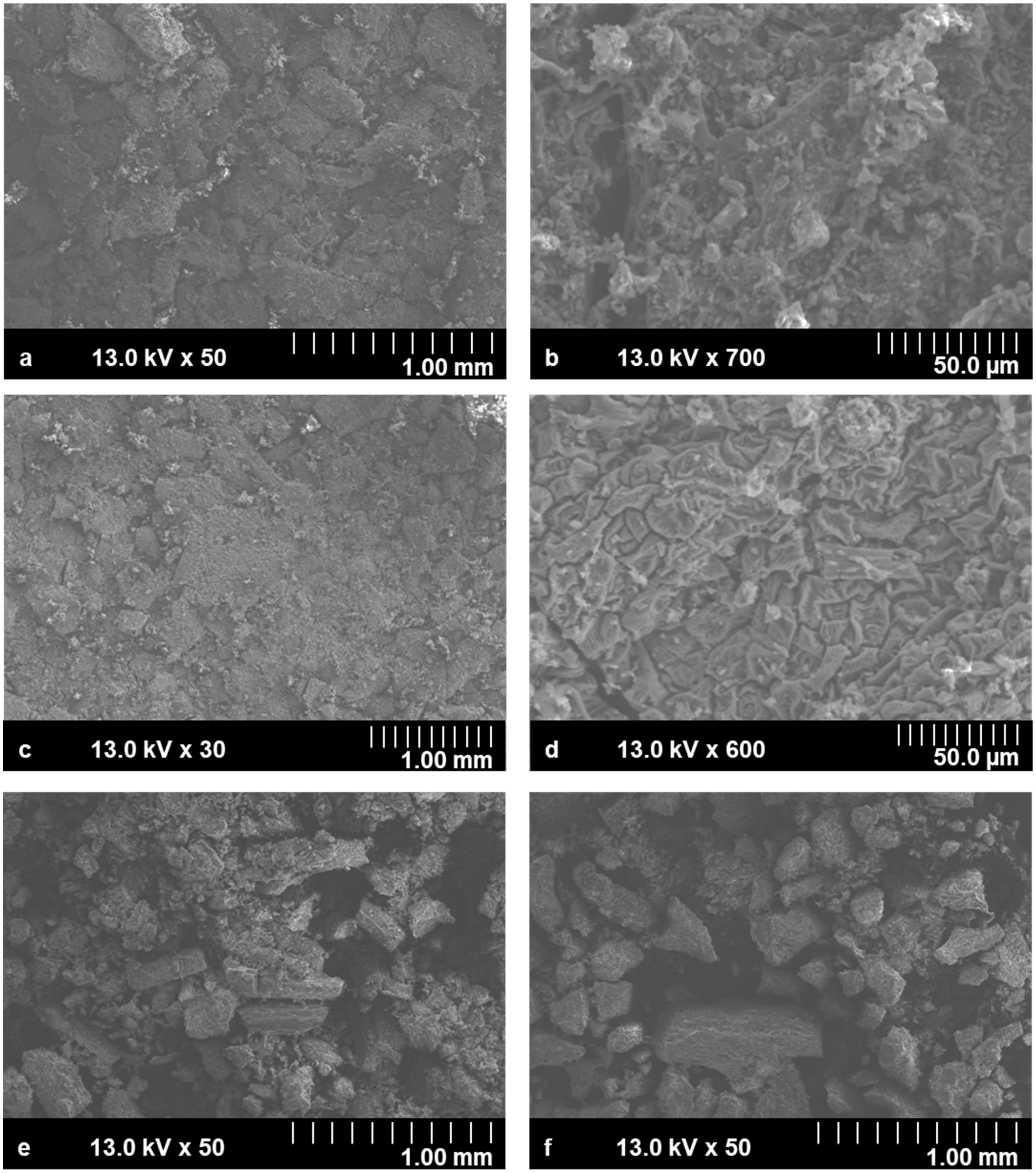

Figure S2. SEM images of biochars with varying initial Ni concentrations: a & b) LLOW-600, c & d) LHIGH-600, e) S0-400, f) SHIGH-400. These images are representative of common biochar structures observed.

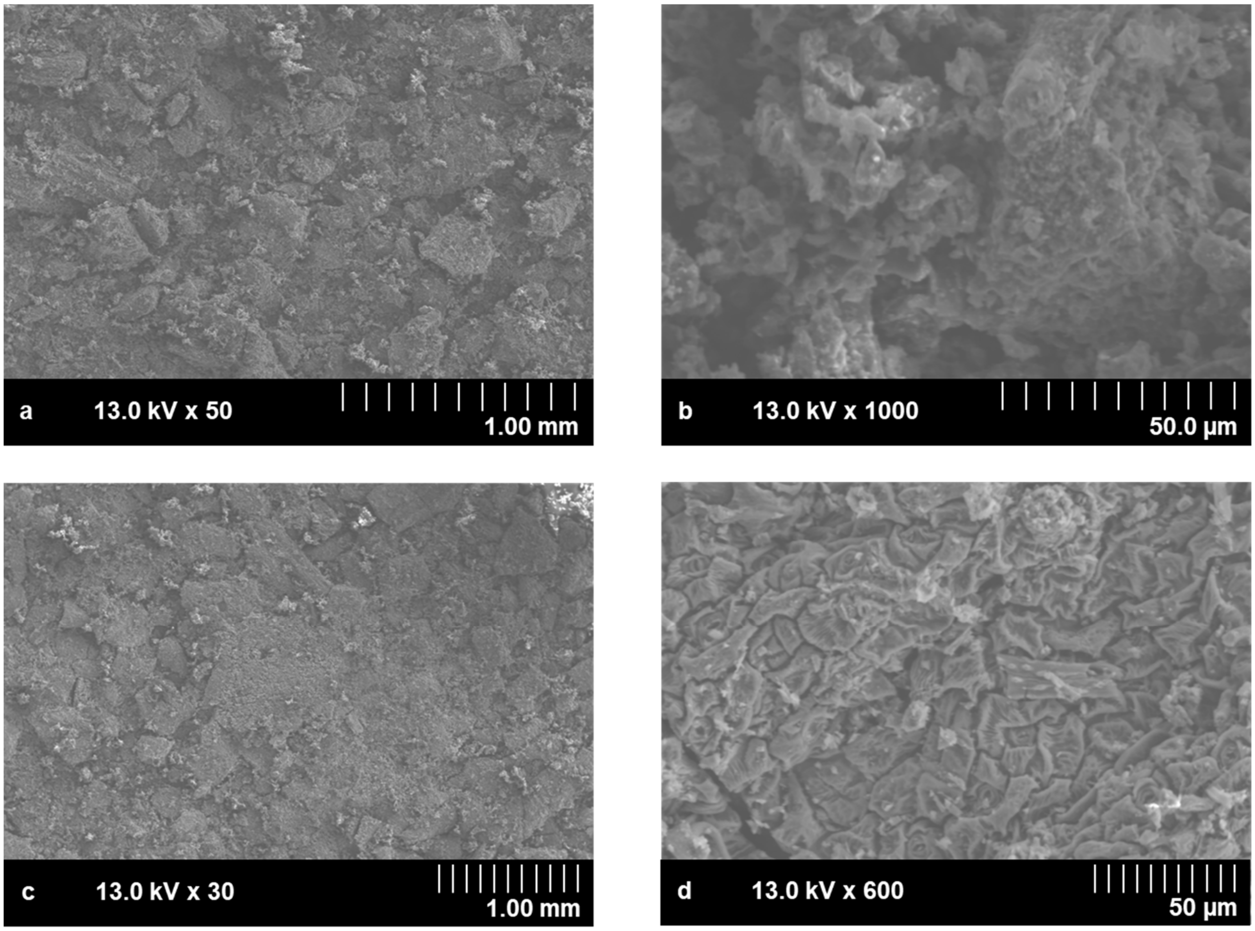

Figure S3. SEM images of aerial plant and leaf biochars at same temperature: a & b) SHIGH-600, c & d) LHIGH-600. These images are representative of common biochar structures observed.

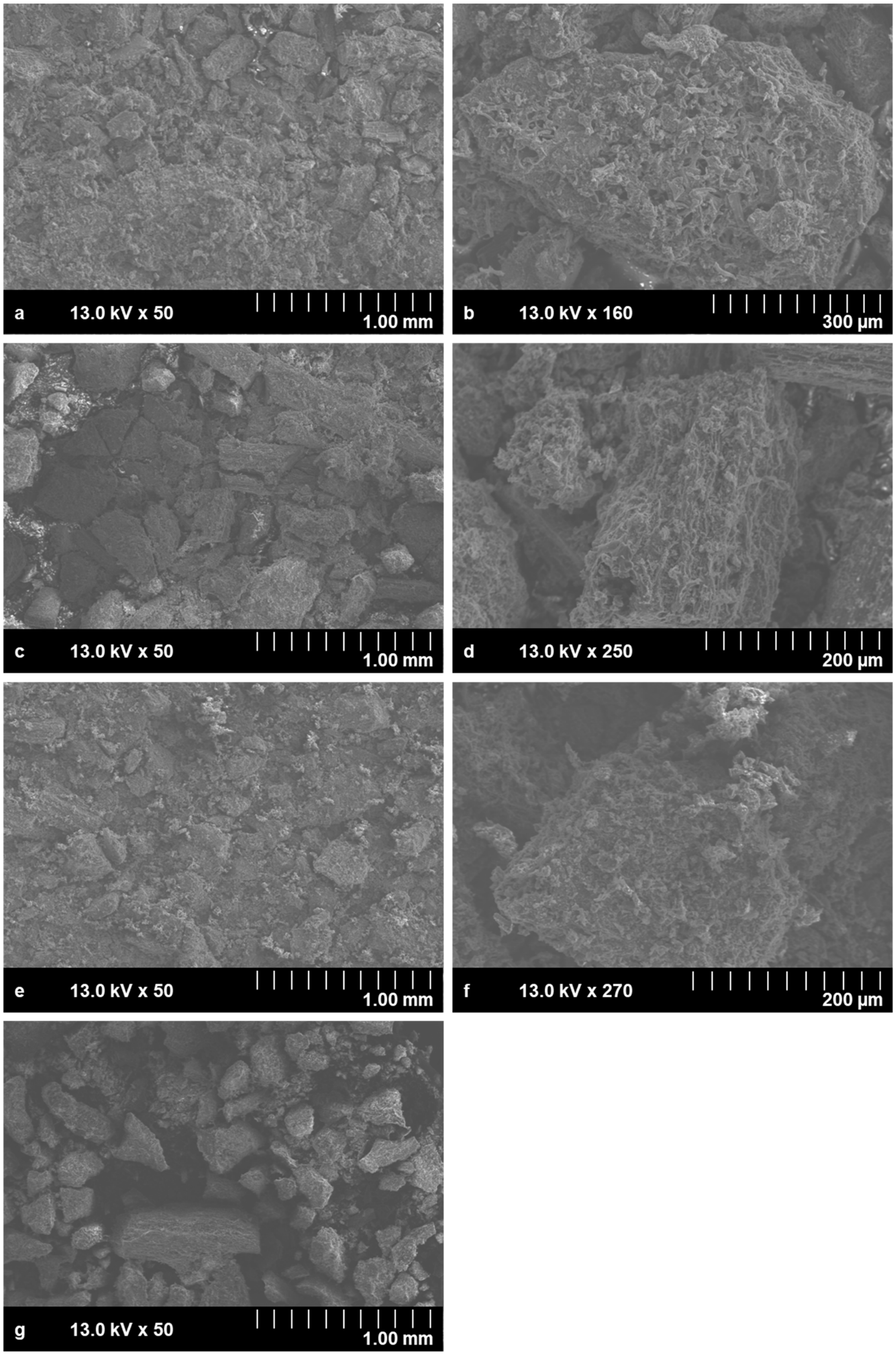

Figure S4. SEM images of the initial plant material SHIGH pyrolyzed at different temperatures: a & b) 900ºC, c & d) 750ºC, e & f) 600ºC, g) 400ºC. These images are representative of common biochar structures observed.

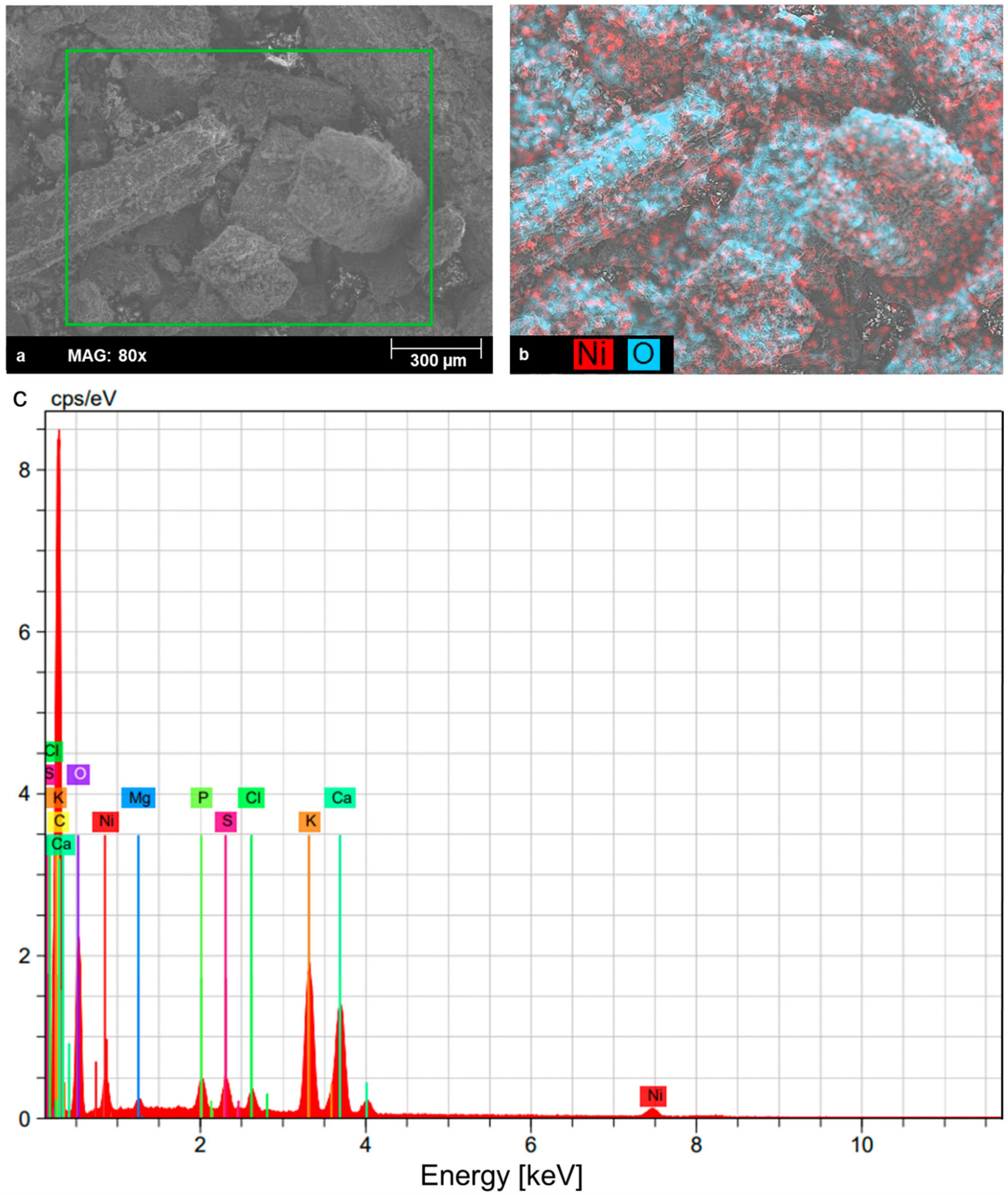

Figure S5. SHIGH-750 a) SEM, b) EDS mapping of Ni and O, c) EDS spectrum of all significant detected elements. The grayscale SEM image in b) is the image in the green box in a) where Ni (red) and O (blue) were mapped. These images are representative of all measured biochar samples.

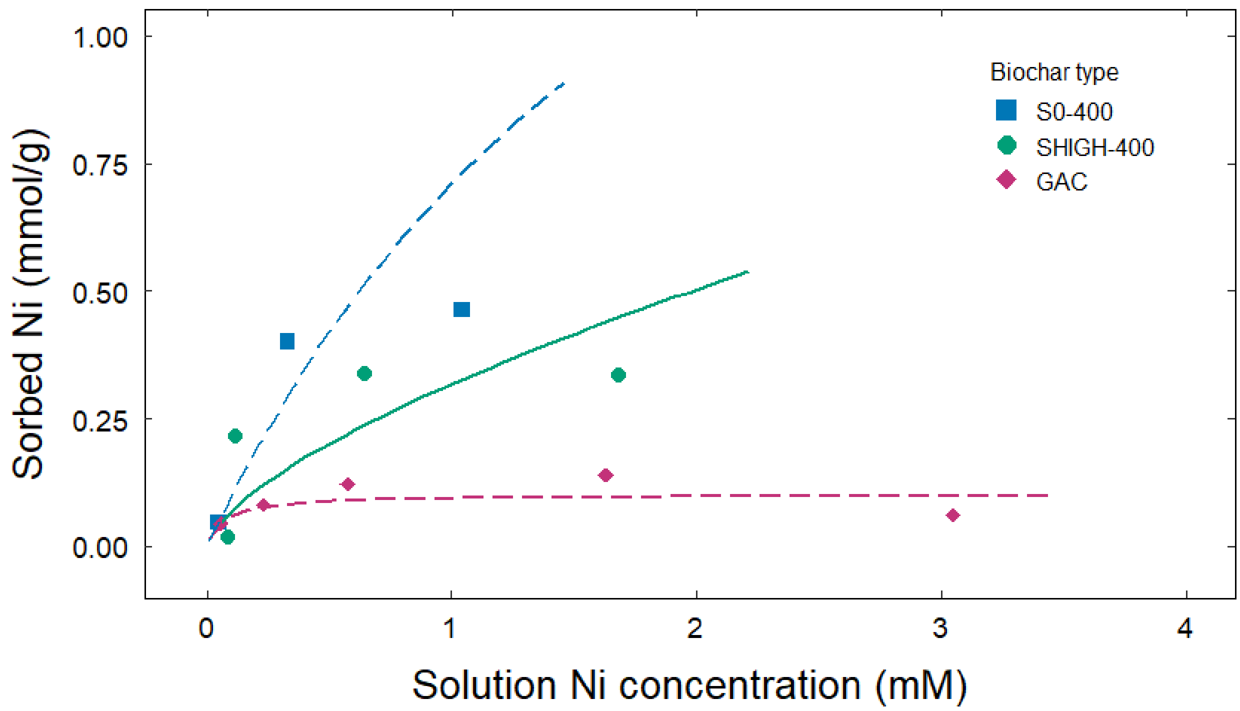

Figure S6. Ni adsorption and adsorption isotherms are plotted. The line color matches the biochar type, and a solid line indicates a Freundlich isotherm while a dashed line indicates a Langmuir isotherm. Only adsorption points with solution Ni concentration > 0 mM were used in the isotherm calculations.
